# *Ammoides pusilla* Essential Oil: A Potent Inhibitor of the Growth of *Fusarium avenaceum* and Its Enniatin Production

**DOI:** 10.3390/molecules26226906

**Published:** 2021-11-16

**Authors:** Yasmine Chakroun, Souheib Oueslati, Vessela Atanasova, Florence Richard-Forget, Manef Abderrabba, Jean-Michel Savoie

**Affiliations:** 1 UR1264 Mycology and Food Safety Research Unit (MycSA), INRAE, F-33882 Villenave d’Ornon, France; yasminechakroun@gmail.com (Y.C.); vessela.atanasova@inrae.fr (V.A.); florence.forget@inrae.fr (F.R.-F.); 2 Laboratory Molecules, Material and Applications (LMMA), Preparatory Institute of Scientific and Technical Studies of Tunis (IPEST), University of Carthage, Sidi Bou Said road, La Marsa B.P. 51 2070, Tunisia; souheibo@yahoo.fr (S.O.); abderrabbamanef@gmail.com (M.A.)

**Keywords:** *Ammoides pusilla*, *Fusarium avenaceum*, mycotoxins, fungistatic activity, volatiles, GC/MS-MS, thymol

## Abstract

Enniatins are mycotoxins produced by *Fusarium* species contaminating cereals and various agricultural commodities. The co-occurrence of these mycotoxins in large quantities with other mycotoxins such as trichothecenes and the possible synergies in toxicity could lead to serious food safety problems. Using the agar dilution method, *Ammoides pusilla* was selected among eight Tunisian plants for the antifungal potential of its essential oil (EO) on *Fusarium avenaceum* mycelial growth and its production of enniatins. Two EO batches were produced and analyzed by GC/MS-MS. Their activities were measured using both contact assays and fumigant tests (estimated IC50 were 0.1 µL·mL^−1^ and 7.6 µL·L^−1^, respectively). The *A. pusilla* EOs and their volatiles inhibited the germination of spores and the mycelial growth, showing a fungistatic but not fungicidal activity. The accumulation of enniatins was also significantly reduced (estimated IC50 were 0.05 µL·mL^−1^ for the contact assays and 4.2 µL·L^−1^ for the fumigation assays). The most active batch of EO was richer in thymol, the main volatile compound found. Thymol used as fumigant showed a potent fungistatic activity but not a significant antimycotoxigenic activity. Overall, our data demonstrated the bioactivity of *A. pusilla* EO and its high potential to control *F. avenaceum* and its enniatins production in agricultural commodities.

## 1. Introduction

Global food safety and sufficiency are facing increasing threats due to climate changes and the consequences on crop production yield, distribution and exposure to pests and diseases [1], to demography growth (United Nations, s.d.) and biodiversity decreases [2]. One of the major hazards that have been threatening food safety during the last decades is mycotoxins contamination [3]. Indeed, mycotoxins are poisonous secondary metabolites with a low molecular weight (0.3–0.7 kDa) and high bioaccumulation ability [4] produced by several filamentous fungal genera such as *Aspergillus*, *Penicillium*, *Alternaria* and *Fusarium* [5,6]. The contamination of crops by these fungi causes an important economic loss and the contamination by their mycotoxins is an important hazard on the animal and human health [7]. Zearalenone (ZEA), type B trichothecenes including deoxynivalenol (DON), nivalenol (NIV) and their acetylated derivatives, ochratoxin A and aflatoxins have been proven to have immunosuppressive, carcinogenic, teratogenic, mutagenic impacts on both animals and humans [5,6,7]. 

Strict measures were implemented to face this hazard worldwide. WHO and FAO have regulated the allowed amount of several of these toxins in multiple food matrixes [8]. Locally, multiple countries have adopted several laws and norms that regulate these occurrences such as EU legislation (EC No 1881/2006) in Europe [9,10]. Furthermore, an increase in the occurrence of “emergent mycotoxins” was recorded worldwide in the last decade. The term includes mycotoxins that are neither routinely monitored nor subjected to regulation [11]. Enniatins (ENNs) are a main group of emergent mycotoxins produced by several *Fusarium* species such as *F. avenaceum*, *F. tricinctum*, *F. acuminatum* that have been detected in various foodstuffs in large amounts [6,11]. Up to now, no evidence of an acute mycotoxicosis has been reported but the increasing frequency of the occurrence of ENNs detected, their bioaccumulation ability and their possible toxicological synergy with other mycotoxins could lead to serious food security concerns. [6,11].

Different strategies are set in place to reduce the *Fusarium* spp. and mycotoxins contamination in the corps worldwide going from the application of different chemical synthetized fungicides to soil tilling and to crop rotation [12]. However, several studies have demonstrated the limits of these strategies because of the deterioration of soil quality and the resistance of the pathogens to the traditional treatments [13]. Face to the global ecological context, the consumer’s awareness has increased, and its alimentation habits had become more natural and bio-oriented [13]. Moreover, the negative effects of chemically-synthetized fungicides on the environment had been proven. Thus, the development of more ecofriendly alternatives has become a major priority. One of the important lines of research is the investigation of the fungicidal potential of essential oils. The use of plant essential oils (EOs) for the elaboration of ecofriendly plant preservatives has various advantages: relatively high volatility, ephemeral biodegradable nature, and consumer tolerance [14]. They are extracted from more than 17,000 aromatic plants belonging to angiosperm families: Lamiaceae, Rutaceae, Myrtaceae, Zingiberaceae and Asteraceae [14], and they have multiple antibacterial, antifungal, antiviral, antiparasitical, insecticidal, medicinal and cosmetic applications [15]. At least 16 different publications [16,17,18,19,20,21,22,23,24,25,26,27,28,29,30,31] presented data on EOs antifungal activities from 35 plants against *F. avenaceum* (Table 1). All the authors used either agar diffusion methods (paper discs or wells), or an agar dilution method. Only one recent paper reported a method for testing the effects of the volatile components [16]. In the application of EOs for controlling the phytopathogens, the bioactivity of volatiles should widely contribute to the fungicidal or fungistatic effect and it is worthy to measure it. However, none of the published work reported the consequences of EO treatments on the production of ENNs by *F. avenaceum*.

Thymol is a component of many essential oils, and it has been shown to be a promising chemosensitizing agent for various pathogenic and opportunistic fungi. It synergistically enhanced the ability of fungicidal formulations intended to inhibit the fungal growth [32] or of citronella and garlic EOs containing monoterpenes and phenols to inhibit *Penicillium corylophilum* [33].

The aim of the present work was to select new EOs having both antifungal and antimycotoxigenic properties. The effect of eight essential oils extracted from Tunisian aromatic plants was evaluated on *F. avenaceum* and its enniatins production. *Ammoides pusilla* EO of thymol chemotype was selected and studied for its composition and activity of its volatiles. Thymol was efficient to limit the growth of *F. avenaceum* but did not inhibit the production of ENNs. *Ammoides pusilla* EO proved to have the potential to control *F. avenaceum* and its production of ENNs.

## 2. Results

### 2.1. Effect of Eight EOs on Mycelial Growth and Production of Enniatins by F. avenaceum

EOs were produced by hydrodistillation of aerial parts from eight Tunisian plants. The extraction yields varied according to the plant species from 0.45 to 2.5% (Table 2). 

**Figure 1:**
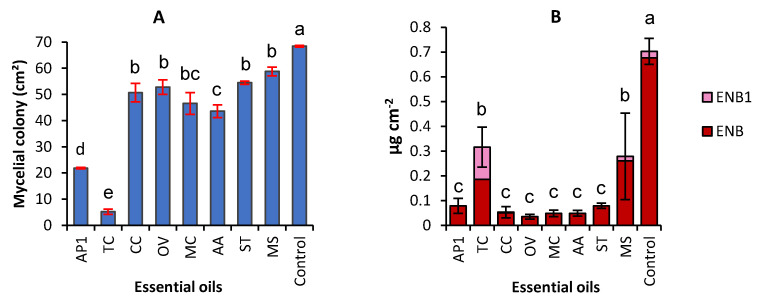
Effect of essential oils extracted from eight Tunisian plants used at 0.1 µL·mL^−1^ on the mycelial growth (**A**) and the accumulation of enniatins (ENNs) (**B**) by *F. avenaceum*. Values are means of 3 replicates. Error bars are ± standard deviation. Means with the same lowercase letter are not significantly different (*p* < 0.05). ENB1 = enniatin B1, ENB = enniatin B. AP1 = *Ammoides pusilla*, TC = *Thymus capitatus*, *CC* = Carum carvi, OV = *Origanum vulgare*, MC = *Myrtus communis*, AA = *Artemisia absintum*, ST = *Schinus terbentofonius*, MS = *Mentha spicata*.

Whatever the considered EO, supplementation of FDM-Agar medium with 0.1 µL·mL^−1^ led to a significant inhibition of *F. avenaceum* mycelial growth (Figure 1A) The inhibition percentages ranged between 14.1% and 92.4%. Two EOs, AP2 and TC, were proved as particularly efficient, with inhibition percentages of 68.1% and 92.4% respectively.

The *F. avenaceum* strain INRA 496 used throughout this study produced enniatin B and B1 but not enniatin A and A1. This production expressed as µg per cm^2^ of the mycelial colony was inhibited by most of the tested EOs at 0.1 µL·ml^−1^ (Figure 1B). MS and TC were the less efficient EOs with an inhibition percentage of 60.4% and 55.1% (ENB + ENB1), respectively. An induction of the production of ENB1 was observed with TC. 

Based on its extraction yield higher than the mean of the other EOs we produced, its significant antifungal activity and its high inhibition of ENN production, *A. pusilla* EO was selected for a more in-depth study on its potential to control *F. avenaceum* and its production of mycotoxins.

### 2.2. Chemical Composition of A. pusilla EO

Two different batches of *A. pusilla* were used to produce EOs; the extraction yields for AP1 and AP2 were 1.60% and 1.64% respectively. Their chemical compositions are reported in Table 3. The main identified components were thymol, γ-terpinene, *p*-cymene and thymol-methyl-ether. They represented 34.70, 27.03, 19.89, 9.18% in AP1 and 53.55, 16.82, 14.59, 8.07% in AP2, respectively. AP2 was more concentrated in oxygenated monoterpenes with thymol accounting for half of the components whereas thymol accounted only for a third in AP1.

### 2.3. Effect of A. pusilla EOs on Mycelial Growth and Production of Enniatins by F. avenaceum

#### 2.3.1. In Vitro Contact Assays in Agar Media

Linear growth indexes were calculated from mycelial areas measured daily for 7 days with different dilutions of AP1 in FDM-agar medium (Table 4). No growth was observed with concentrations equal or higher than 0.75 µL·mL^−1^ of AP1 EO, and an index close to 0 at 0.5 µL·mL^−1^ was determined. The minimum inhibitory concentration (MIC), defined as the lowest concentration of the EO which completely prevented visible fungal growth, was estimated to be nearly 0.5 µL·mL^−1^. At this concentration, a 99.2% inhibition of fungal growth was observed after 7 days of incubation. Using a linear regression of the data in Table 4, excluding 0.75 and 1 µL·mL^−1^ (R^2^ = 0.92), the concentration leading to 50% growth inhibition (IC50) was calculated to be 0.23 µL·mL^−1^. The accumulation of ENNs per unit of mycelial area was inhibited by 65, 76 and 100% at AP1 concentrations of 0.1, 0.25 and 0.5 µL·mL^−1^, respectively. Using a linear regression (R^2^ = 0.91), IC50 of ENNs accumulation was estimated equal to 0.12 µL·mL^−1^.

When considering AP2, MIC and IC50 calculated for 10-day old cultures were 0.25 µL·mL^−1^ and 0.10 µL·mL^−1^ (R^2^ = 0.99) respectively. A 92% reduction of ENN accumulation was induced by a concentration of 0.15 µL·mL^−1^ of AP2 while this inhibition percentage was 57% at 0.05 µL·mL^−1^. Using a linear regression (R^2^ = 0.99), IC50 for the reduction of ENN accumulation was estimated to be 0.048 µL·mL^−1^. Altogether, the previous data support the high inhibitory efficiency of *A. pusilla* EO, AP2 being more than 2-fold more effective than AP1.

#### 2.3.2. Effect of Volatiles Components

**Figure 2:**
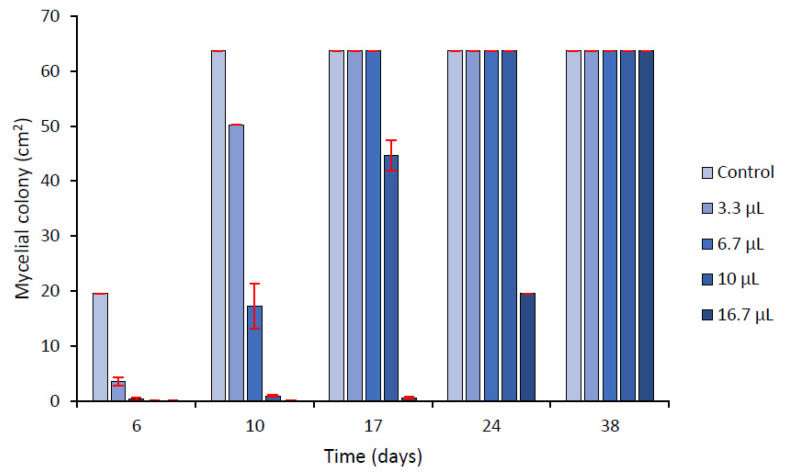
Mycelial growth of *F. avenaceum* in presence of different quantities of volatile compounds of *A. pusilla* EO (AP1). The volumes in µL are the quantity of EO per L of air. Means of 3 replicates. Errors bars are ± standard deviations.

Fumigation assays have evidenced significant antifungal effects of the volatiles contained in AP EOs. Increasing quantities of EO led to slow-down of fungal growth kinetics, due to increased lag phases and decreased of linear growth indexes. In control conditions, the maximum area (full invasion of the plates) was reached after 10 days of incubation. With 16.7 µL·L^−1^ of EO diffusing in a closed jar, an important fungal growth reduction was observed up to 17 days. After 38 days, the mycelium reached the border of the plates (Figure 2).

Linear growth index until 24 days and inhibition percentages calculated at day 10 are presented in Table 5. The IC50 value determined using a linear regression was 7.58 µL·L^−1^. Volatile compounds were also shown to induce a significant inhibition of ENN accumulation (Table 5). Using a linear regression (R^2^ = 0.97), IC50 of ENN accumulation was estimated to be 4.2 µL·L^−1^.

**Figure 3:**
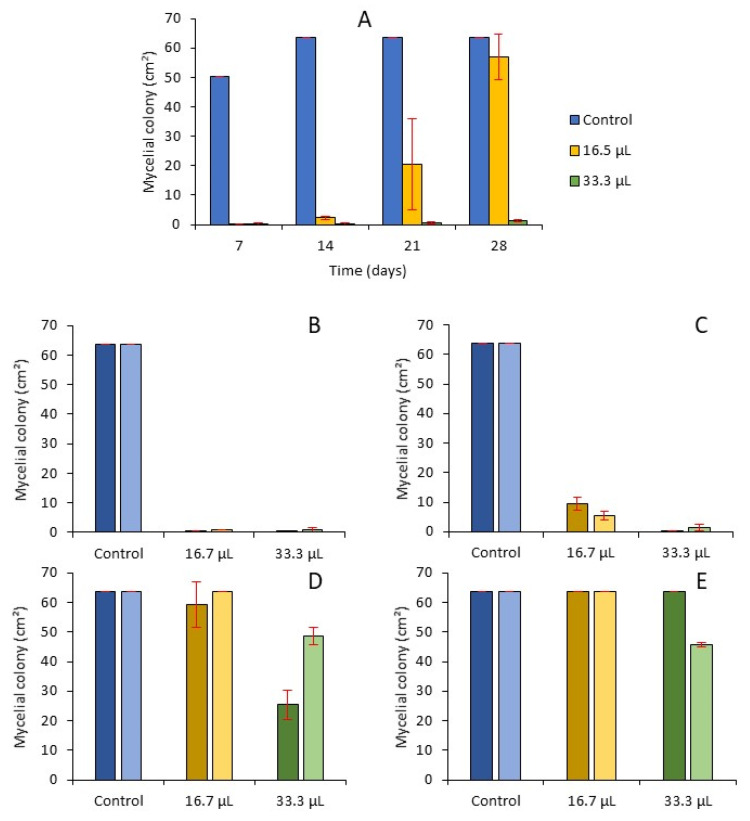
Mycelial growth of *F. avenaceum* in presence of volatile compounds from 16.7 or 33.3 µL EO L^−1^ in consecutive cultures. (**A**) First exposure to volatiles. (**B**,**C**) second exposure with the same quantity of EO as in the first exposure of fungal material coming from (**A**) as spore suspension (dark bars) or plugs (light bars) (second culture-EO). (**D**,**E**) second culture without EO of fungal material coming from (**A**) as spore suspension (dark bars) or plugs (light bars); µL are those of the first exposure (second culture-control). (**B**,**D**) are measures at 10 days, (**C**,**E**) are measures at 28 days. Means of 3 replicates. Error bars are ± standard deviations.

The behavior of the mycelial growth over a long exposure was assessed with a second assay using two concentrations of EO (16.66 and 33.3 µL·L^−1^) for 28 days (Figure 3A). The inhibition recorded at day 21 with 16.7 µL·L^−1^ (68%) was not different from the value recorded at day 24 in the previous assay (Figure 2) whereas a non-significant inhibition (10%) was recorded at day 28. Using the highest quantity of EO (33.3 µL·L^−1^), 98% of mycelial growth was still inhibited at day 28.

**Figure 4:**
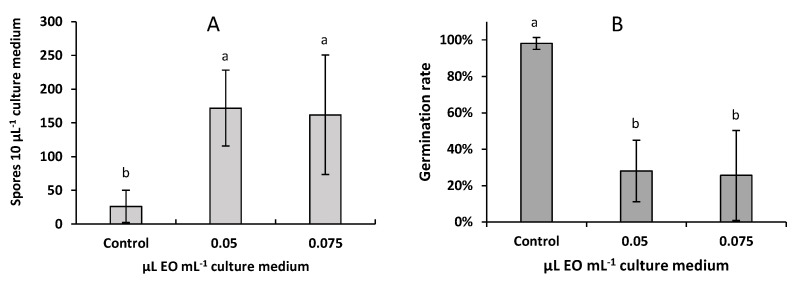
Changes in sporulation capacity (**A**) and rate of spore germination (**B**) after treatment with two concentrations of *A. pusilla* EO for 24 h. Means of 3 replicates. Errors bars are ± standard deviations. Means with the same lowercase letter are not significantly different (*p* < 0.05).

After this first 28 day-long exposure time to EO volatile components (16.7 or 33.3 µL EO L^−1^), either plugs or spores were collected and used for inoculation of new plates placed either under the same conditions as the controls without EO (second culture-control) or in the presence of the volatiles in the same quantity of EO than in the first culture (second culture-EO). The second culture-control recovered their ability to grow, with a low remaining inhibition effect for the exposure to volatiles from 33.3 µL EO L^−1^, whatever plugs or spore suspensions were used as inoculum (Figure 3D,E). In the case of the second culture-EO, the inhibitory effects were improved (Figure 4B,C). At 28 days, during the second exposure at 16.7 µL EO L^−1^ for 28 days, the mycelial surface was 83% lesser than in the first exposure.

### 2.4. Effect of A. pusilla EO on the Sporulation and Spore Germination of F. avenaceum

As shown in Figure 4, the ratio of germinated spores after contact of *F. avenaceum* for 24 h with 0.05 or 0.075 µL *A. pusilla* EO L^−1^ decreased significantly compared to controls. On the opposite side, the exposure to the EO increased spore production (Figure 4).

### 2.5. Antifungal and Antimycotoxigenic Activity of Thymol on F. avenaceum

**Figure 5:**
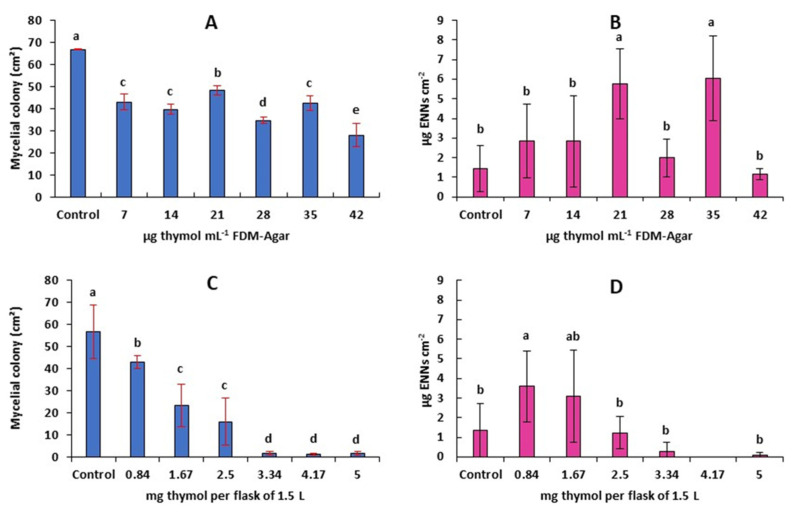
Effect of thymol on the mycelial growth rate (**A**,**C**) and production of ENNs (**B**,**D**) by *F. avenaceum*. Thymol was either diluted in the culture medium (**A**,**B**) or use as a fumigant (**C**,**D**). Values are means of 6 replicates. Error bars are ± standard deviation. Means with the same lowercase letter are not significantly different (*p* < 0.05).

As thymol was shown to be the main component of *A. pusilla* EO, we tested its biological activity against *F. avenaceum*. When diluted in the agar medium, a significant inhibition (25%) of the mycelial growth was observed at the lowest concentration tested, 7 µg·mL^−1^. This inhibition did not increase with higher concentrations (Figure 5A). When thymol was used as a fumigant (Figure 5C), a dose-dependent activity was observed. Both with *in vitro* contact assay and fumigation assay, stimulation of ENNs production was observed in several samples but none of the decreases in concentrations due to treatment was significant (Figure 5B,D).

## 3. Discussion

In order to find an EO with a high potential for inhibiting both the mycelial growth and ENN production by *F. avenaceum*, eight different Tunisian aromatic plants were collected and their EOs were produced by hydrodistillation. All EOs were efficient for limiting the mycelial growth with different levels of antifungal activity. EOs of four of the species had been previously tested for their antifungal activity against *F. avenaceum*: *C. carvi*, *M. spicata*, *O. vulgare*, *T. capitatus* [16,17,22,25,30]. Due to the different ways in which the antifungal activities were measured and reported in the different previous works (see Table 1), it was difficult to compare the efficiency of the EOs. *Carum carvi* EO was one of the two most effective among the 6 commercial EOs tested by Paškevičius et al. [17] whereas a low activity was observed here. For *O. vulgaris* EO, the previous work reported MICs of 0.08 to 0.25 µL·mL^−1^ [16,25] and 77% inhibition of the mycelial growth at 0.5 mg·mL^−1^ [22]. In the same study *T. capitatus* EO inhibited up to 90% of the mycelial growth [22]. In the present study with a lower concentration (0.1 µL·mL^−1^) the *T. capitatus* EO had the highest antifungal activity (92% inhibition).

To the best of our knowledge, this article shows for the first time the antifungal activity against *F. avenaceum* of EOs extracted from *A. absintum*, *M. communis*, *S. terbentofonius* and *A. pusilla*. In a recent overview of the published studies on the antifungal properties of EOs, these four species were not listed [34]. *Ammoides pusilla* EO was the second-best EO for inhibiting mycelial growth and is a candidate, along with *T. capitatus* EO, for the control of *F. avenaceum* in cereals.

Mycotoxin production was suspected to be part of the adaptive response of fungi to stressful conditions such as an exposure to antifungal compounds [35,36]. It was commonly observed with molecules, such as phenolic acids, active on both fungal growth and mycotoxin production by *Fusarium* species that the fungal response is strain and concentration-dependent. For instance, ferulic acid can either activate the production of *Fusarium* mycotoxins at low concentrations or inhibit them at higher concentrations [37,38,39]. No correlation between antifungal and antimycotoxigenic effects was also observed with some EOs. In maize samples inoculated with *F. graminearum*, treatment with Fennel EO greatly reduced the fungal biomass (81%) as assessed by the amount of ergosterol in the grains, while no more than a 7% decrease in ZEA concentration was found [40]. This suggested an induction of ZEA production in the remaining mycelium in very unsuitable growth conditions. To select effective EOs, the antimycotoxigenic activities or at least the absence of risk of induction of the production of mycotoxins under certain conditions must also be studied. It is interesting to note that all EOs tested in the present work significantly inhibited the production of ENNs by *F. avenaceum*. These activities had not been reported before. Both EOs from *T capitatus* and *M. spicata* were less efficient. Consequently, *A. pusilla* EO appeared as the best candidate to control *F. avenaceum* and its ENNs production.

*Ammoides pusilla* (Brot.) Breistr. is a halophilic annual herbaceous *Apiaceae*, that is found widely in arid and semi-arid Mediterranean regions and is used as a traditional medicine [41]. The extraction yield of EOs and their composition depended on the plant part used, the extraction method, the plant stage of development, the harvesting season and the geographical origin. Eight plants of *A. pusilla* from eight Algerian localities showed that flower parts gave higher percentages of extraction by hydrodistillation than leaf parts [42]. Oxygenated monoterpenes were the main components of the *A. pusilla* EOs, except for the EO extracted from leaves originated from Ain Kihal and the EO extracted from the flower part originated from Fehoul [43]. In a previous study, the same authors found 57% of oxygenated terpenes in aerial part EO [39]. A significant antioxidant activity of *A. pusilla* EO has been attributed to their high content in phenolic terpenes, especially thymol [41,42].

In the present study *A. pusilla* plants were collected fresh in Tunisia at the flowering stage and air-dried (AP1) or purchased as dry material on a market (AP2). The yields of EO extraction agreed with a previous work reporting that aerial parts of Tunisian *A. pusilla* yielded 1.6% EO by hydrodistillation and leaves and flowers were more productive (2.7%) [43]. The composition of the two EOs we obtained differed. Oxygenated terpenes were dominant (66.6%) in AP2 as in [41] whereas monoterpene hydrocarbons dominated (48.8%) in AP1. The composition of AP1 was close to that of the EO studied by Souhaiel et al. [43] in which the main components for the aerial parts were thymol (33.0%), γ-terpinene (28.2%), *p*-cymene (15.3%) and thymol-methyl-ether (8.9%). The difference in composition between AP1 and AP2 is a new example of variation in composition with the batch of plants used for EO extraction. However, both EOs were of the thymol chemotype, as in most published compositions of *A. pusilla* EOs, although in rare studies thymol was not the main component [41].

The difference in EOs’ chemical composition may affect their antifungal activity. Using dilutions of EOs in an agar medium, the MIC and the IC50 of AP2 were two times lower than those of AP1. Thymol was the main component of the EOs accounting for 53.6% and 34.7% of volatile components in AP2 and AP1 respectively. This result led us to propose that the higher activity of AP2 was due to its higher concentration in thymol. This phenol is known to have strong antifungal activities on twelve different strains of mycotoxigenic fungi, belonging to different species and involved in several plant diseases [44]. We observed it was active also against *F. avenaceum*, both in agar dilution assay or as a fumigant. Its hydrophobicity allows it to accumulate in the lipids of the cell membranes reacting with cell proteins and finally drastically affecting the membrane integrity [45]. Thymol functions also as a redox-active compound, inducing generation of reactive oxygen species and lipid peroxidation in fungal cells [46] and inhibiting genes involved in cell membranes synthesis [47]. In this way, it acts as a chemosensitizing agent for various pathogenic and opportunistic fungi [32,33]. Thus, the richness of *A. pusilla* EO in thymol acting in synergy with other components may be at the origin of its antifungal potential.

No negative effect of *A. pusilla* EO was observed on the sporulation rate of *F. avenaceum*, in contrast to the 100% inhibition recorded with a thyme EO of thymol chemotype diluted at 0.04 µL·mL^−1^ [25] and the significant effect of thymol on *F. verticillioides* [48] 

We measured the antifungal activity of the *A. pusilla* EO against *F. avenaceum* after inoculation of an agar medium with spore suspensions placed at the center of the Petri dishes. The inhibitions we measured finally resulted from both negative effects on the germination of spores and decreases in the mycelial growth rates. We measured that *Ammoides pusill*a EO diluted at 0.05 µL EO mL^−1^ inhibited 71% of conidia germination. It was previously reported that thymol was effective against *Fusarium* spp conidia germination. For instance, 0.25 mg ml^−1^ inhibited 100% of germination of *Fusarium oxysporum* f. sp. *dianthiafter* conidia after contact for 24 h [49], and 1 mM inhibited 81% germination of *F. verticillioides* conidia after contact for 12 h [48]. Furthermore, volatiles of commercial EOs of cinnamon, citronella, clove, lemongrass, oregano, thyme inhibited the sporulation of *F. avenaceum* at rates ranging from 71 to 100% [16]. Among the tested commercial EOs, an oregano EO of carvacrol chemotype was the least efficient and a thyme of linalool chemotype was the most potent one [16].

Actually, testing the effect of volatile compounds identifies indirect activity of EOs as opposed to direct contact in the agar dilution methods in which non-volatile components may contribute to the overall antifungal effect [15]. Surprisingly, the bioactivity of EO volatiles on *F. avenaceum* had been tested in only one recent paper (Table 1). In this study, 10 µL of pure EO was added to an inverted Petri dish [16], which resulted in higher concentrations of volatiles than in the present work where 3 to 33 µL EO L^−1^ air were used. In addition, the colony diameters were measured after 10 days while incubations lasted 24–28 days in our study. Consequently, we did not observe the fungicidal effect reported for all the EOs tested by Parikh et al. [16], but only fungistatic effects. Furthermore, EO concentrations leading to 100% inhibition during the first two weeks did not block but only slowed down the mycelial growth with a longer exposure (38 days). This may be attributed to the decrease in concentrations of active compounds over time due to EO degradation that was responsible for the loss of inhibition activity. EOs are known to be sensitive to conversion and degradation reactions modulated by temperature, light, and oxygen availability [24,50]. These effects on mycelial growth tended to disappear after transfer of both mycelium and spores to fresh agar medium under fresh air without EO vapors, but they were amplified when the transfer was made under conditions where volatiles from a new EO application were provided. There were no residual properties of EO and maintaining exposure to concentrations of EO volatiles having a fungistatic effect would be a prerequisite from the use of *A pusilla* EO to control *F. avenaceum*.

Clearly, it is important to ensure that any antifungal compound used to control *F. avenaceum* provides no significant induction of ENN production when applied at doses below the lowest concentration inhibiting 100% of mycelial growth. Thymol and other redox-active compounds inducing oxidative stress were reported to activate the biosynthesis of trichothecenes production in some *Fusarium* species [35,51]. However, in other studies treatment of fungal cultures by thymol did not boost DON and ZEA production and did not up-regulate key toxin biosynthetic genes in *F. culmorum*, at the concentration used [32]. Overall, treatments having both antifungal and antimycotoxigenic effects are expected when searching for a potent EO. This was the case here with *A. pusilla* EO. In contact assays, the IC50 for ENN accumulation was half the IC50 for mycelial growth. The volatiles compounds from two amounts of EOs inhibiting 22 and 73% of the mycelial growth and decreased the concentration of ENNs by 67 and 80%, respectively. Hydroxycinnamic acids were shown to interfere with both the growth of *F. avenaceum* and its production of ENNs [52]. However, in certain situations, a significant reduction in ENNs yield and a drastic transcriptional down-regulation of two genes involved in ENNs biosynthesis were observed while *F. avenaceum* growth was not or weakly affected, corroborating the occurrence of a regulatory mechanism that specifically targeted the production of ENNs [52]. With *A. pusilla* EO also, the antifungal and antimycotoxigenic activities could be partly the results of independent regulation mechanisms that need to be elucidated. Nothing is known regarding the effect of oxidative stress on the production of ENNs, but as proposed for ferulic acid [52], the capacity of the EO to alleviate oxidative stress [38,39] could explain its inhibiting activity on ENNs production.

This first investigation on the antimycotoxigenic effect of *A. pusilla* EO had shown that this EO could be used to decrease the accumulation of ENNs by *F avenaceum* in cereals, ensuring improved food security for this sector.

## 4. Materials and Methods

### 4.1. Plant Material and EOs 

*Ammoides pusilla* (AP_2_), *Thymus capitatus* (TC), *Carum carvi* (CC), *Origanum vulgare* (OVEO), *Myrtus communis* (MC), *Artemisia absintum* (AA) were purchased from a local medicinal plant market in Tunisia at a dry state of the plant aerial parts. *Mentha spicata* aerial part (MS), *Schinus terbenthifolius* fruits and leaves (ST) and a second lot of *Ammoides pusilla*, aerial parts at the flowering stage (AP_1_) were gathered by LMMA laboratory members, air-dried under dark conditions. The samples were stored under dry and dark conditions before extraction of EOs. EOs extracted from these plant batches had previously been tested for their potential bioactivities [43,53].

All the essential oils were extracted through hydrodistillation using a conventional glass Clevenger-type apparatus. Anhydrous sodium sulfate (Na_2_SO_4_) was added to the extracted oils to eliminate eventual residual water. The extraction yield was measured as the percentage of oil weight based on the dry plant material weight, following the equation: EY = EOW/DMW × 100 where EY: extraction yield, EOW: essential oil weight, DMW: dry plant material weight. The essential oils were stored at 4 °C until being used. 

### 4.2. Fusarium Strain and Media

The strain of *F. avenaceum* I496 used throughout this study has been isolated from maize in France, in 2007, and stored in the fungal collection of the French National Research Institute for Agriculture, Food and Environment (INRAE/MycSA collection). A stock culture maintained at 4 °C on potato dextrose agar (PDA) slants under mineral oil was sub-cultivated on PDA medium in 55 mm Petri dishes and kept at 4 °C until being used in the different tests. 

When inoculum was required, *F. avenaceum* was grown on PDA plates for 3–5 days at 25 °C, and conidia suspensions (10^6^ conidia mL^−1^) were prepared as in Gautier et al. [52]. 

Assays on growth inhibition and production of mycotoxins were performed on FDM-Agar medium in Petri dishes (diameter 90 mm) supplemented with EOs and inoculated at the center with 10 µL of conidia suspension or alternatively with an agar plug from a previous culture on FDM-Agar. FDM is a synthetic medium promoting the production of ENNs by *F. avenaceum* [54]. The EOs were diluted in ethanol and added to the FDM-agar culture medium cooled to 55 °C (still liquid) after autoclaving, stirred for 5 min and poured into 9 cm diameter Petri dishes, 10 mL per plate. Controls with diluting solvent were prepared.

### 4.3. Agar Dilution Assays 

#### 4.3.1. Comparison of Eight EOs

The EOs were diluted at 0.1 µL·mL^−1^ FDM-Agar medium and the plates were inoculated with conidia suspensions. The plates containing the different essential oils and the control were put in different hermetically sealed preservation jars with an internal volume of 1.5 L and incubated at 25 °C for 10 days. Mycelial growth was assessed at day 5 and day 10 after inoculation by measuring the area covered by the mycelial colony on the plates using pictures analyzed with the ImageJ software. The inhibition rates were calculated by comparisons with controls without EO. Three plates were measured at each incubation time for each EO. Immediately after taking the picture, the plates were stored at −20 °C before extraction and qualification of ENNs.

#### 4.3.2. Activity of *A. pusilla* EO on *F. avenaceum* in Contact Assay

The MIC (Minimum Inhibitory Concentration) is defined as the minimum concentration that inhibits 100% of the mycelial growth and the IC50 as the concentration that inhibits 50% of the mycelial growth. Two different extractions of *A. pusilla* EO were used: AP1 and AP2. AP2 was diluted in the FDM-Agar medium at 0 (control), 0.05, 0.1, 0.15, 0.2, 0.25 and 0.5 µL·mL^−1^ and AP1 dilutions were 0, 0.1, 0.25, 0.5, 0.75, 1, 2 and 5 µL·mL^−1^. Each treatment was triplicated and each set of triplicates was incubated in different hermetically closed jars at 25 °C for 10 days. Pictures of the plates were taken every 2 days and the areas covered by the mycelial colonies measured. The growth index of *F. avenaceum* at each concentration was calculated as in Krzyśko-Łupicka et al. [55]: T = A/D + b1/d1 + … + bx/dx, where: T= index of linear growth; A = average measurement value of mycelial surfaces (cm^2^); D = duration of the experiment; b1 … bx = mycelial surfaces (cm^2^); d1 … dx = number of days since last measurement. Straight linear regression equations defining the relationship between the decimal logarithms of EO concentrations and the growth index or the mycelial area at the end of the experiment (10 days) were used to calculate the IC50. On day 10, the plates were conserved at −20 °C until their use for ENNs analysis of ENNs.

### 4.4. Effect of Volatile Compounds of A. pusilla EO on F. avenaceum

The evaluation of the effect of the volatile compounds of *A. pusilla* EO was performed in fumigation assays by the exposure of *F. avenaceum* to a set of concentration of the AP2: 0 (control), 3.33, 6.66, 10 and 16.66 µL EO mL^−1^ of air volume. 

Petri dishes (90 mm) were marked with two perpendicular length scales. For each concentration, sets of three dishes were filled with 10 mL of FDM-agar medium and inoculated at the center part with 10^4^ spores of *F. avenaceum*. The dishes without lids were put into preservation jars (3 dishes in each) of 1.5 L. One cm^2^ of sterile filter paper was put in the center of open 55-mm Petri dishes. The appropriate amounts of the EO were deposited onto the filter papers, one Petri dish was put into each jar, and the jars were hermetically closed. The jars were incubated at 25 °C for 10 or 24 days. The mycelial growth was evaluated at 6, 10, 17 and 24 d of incubation thanks to the length rules without destruction of the dishes nor disturbing the incubation in jars. The mycotoxins were extracted in the culture medium of 3 dishes from a jar per concentration after incubation for 10 and 24 days.

In order to evaluate the persistence of the effect of a first treatment by the volatile compounds of AP_2_ EO, two concentrations having high activities were selected: 16.67 and 33.33 µL·L^−1^ air. Each treatment and control without EO were triplicated. The jars were incubated at 25 °C for 28 days and the mycelial growth was measured every week as above until 28 days of incubation at 25 °C. On day 28, either plugs (diameter 0.5 cm) or spores were collected from the mycelial colonies. Either a mycelial plug or 10^4^ spores were reinoculated on new Petri dishes with FDM-agar culture medium. The Petri dishes with FDM-Agar culture medium inoculated with spores or plugs originating from the first exposure at 16.67 or 33.33 µL·L^−1^ were incubated at 25 °C for 28 days in presence of 16.67 or 33.33 µL L^−1^ AP_2_ EO respectively, and in absence of EO. The Petri dishes with FDM-agar culture medium inoculated with spores or plugs originating from the first control without EO were incubated without EO. 

### 4.5. Effect A. pusilla EO on Production and Germination of Conidia by F. avenaceum

To study the effect of *A. pusilla* EO on conidia production and their ability to germinate, suspensions of *F. avenaceum* conidia were prepared as in 4.2 and used to inoculate CMC liquid medium. The cultures were incubated for 3 days at 25 °C, and EO was added at two concentrations, 0.05 µL and 0.075 µL EO mL^−1^. In the control, 2% of ethanol was added. After incubation for 24 h, the cultures were thoroughly mixed and three aliquots of suspension were filtered and collected. The number of total conidia in 10 µL of suspension was recorded under a microscope. The conidia suspensions were incubated for 24 h at 25 °C in liquid FDM medium and 10 µL were observed under a microscope to record the percentage of germinated conidia. 

### 4.6. Effect of Thymol on F. avenaceum

Pure thymol, 5-methyl-2-isopropylphenol (Sigma-Aldrich, Saint-Quentin-Fallavier, France) was used both in agar dilution assays and in fumigation as for the EO. Conidia suspensions were inoculated, and incubation set for 10 days at 25 °C. In the agar dilutions assays, the concentrations used were 7, 14, 21, 28, 34, 43 µg mL^−1^. In the fumigation assays, they were 0.84, 1.67, 2.5, 3.34, 4.17, 5 mg L^−1^.

### 4.7. Chemical Composition of A. pusilla EO

Gas chromatography-mass spectrometry (GC–MS) analysis of EOs was performed on an Agilent 7890 gas chromatograph, coupled to an Agilent 5975C mass spectrometer with electron impact ionization (70 eV) (Agilent Technologies, Santa Clara, CA, USA), using an HP-5 column (30 m × 0.25 mm, film thickness 0.25 μm). The operating conditions for gas chromatography analysis were as follows: the detector temperature 280 °C; the injector temperature 210 °C; the oven temperature program varied from 180 °C (1 min) to 200 °C (15 min) then from 220 °C (3 min) to 300 °C (10 min). Carrier gas was H_2_ (0.9 mL min^−1^); split ratio of 1:100 and a linear velocity of 36.45 cm s^−1^ was used as carrier gas and mass spectral range was recorded from m/z 50–550 amu. The injected volume was 1 μL of 1% EO solution in hexane (Purity ≥ 97%) (Merck, Darmstadt, Germany). The identities of the EO components were established by comparison of their MS spectra with those reported in literature and by computer matching with the Wiely mass spectra library (Eleventh Edition/NIST 2009).

### 4.8. Extraction and Quantification of Enniatins

The content of a Petri dish (agar medium plus mycelium) was sliced and placed in 35 mL of ethyl acetate (VWR, Fontenay-Sous-Bois, France) for 15 min at room temperature with agitation at 250 rpm. After filtration on n°4 filter paper, 5 mL were evaporated to dryness at 45 °C under nitrogen flow. The dried samples were dissolved in 200 µL of methanol/water (1:1, *v*/*v*) and filtered on 0.2 µm filters before analysis. Quantification of ENNs was performed on a Shimadzu Prominence UPLC chain, equipped with two pumps LC-30 AD, a degasser DGU-20A5R, an auto sampler SIL-30 AC and a DAD detector SPD-M20A (Shimadzu Scientific Instruments, Noisiel, France). Separation of 5 µL of extract was achieved on a Kinetex 2,6U XB-C18—100 Å column (150 × 4.6 mm; 2.6 µm) (Phenomenex, Le Pecq, France) maintained at 45 °C. An elution gradient of acetonitrile in water was used with a constant flow at 1.4 mL min^−1^: 30% acetonitrile for 2.5 min, 30–99% acetonitrile in 5 min, 99% acetonitrile for 3.5 min, and 2.5 min post-run equilibration with 30% acetonitrile. LC-grade methanol and acetonitrile were purchased from VWR (Fontenay-Sous-Bois, France). Absorbance spectra were recorded from 190 to 450 nm and peak areas were measured at 205 nm. External calibration with standard solutions of A, A1, B and B1 enniatins (Sigma-Aldrich, Saint-Quentin-Fallavier, France), allowed quantifications between 1 and 100 µg·mL^−1^. The limit of quantification was 1 µg·mL^−1^.

### 4.9. Statistical Analyses

Results were given as mean values ± standard deviation of three biological replications. Mycelial growth parameters and ENNs concentrations were subjected to ANOVA and differences between treatments were tested with Duncan’s multiple range test. Student test (comparing the result of each condition with its respective control was used for the sporulation capacity and rate of spore germination. All the statistical analysis was performed using SAS Software (Statistical Analysis System, version 9, Cary, NC, USA). *p* values < 0.05 were regarded as significant.

## 5. Conclusions

The present study evidenced for the first time the capacity of essential oils and their volatile parts to efficiently reduce the production of ENNs by a *F. avenaceum* strain, in addition to fungistatic activities. The data obtained with *A. pusilla* EO and pure thymol confirmed the contribution of thymol to the efficiency of the EO, but not to the EO capacity to reduce the production of ENNs. This supports the interest of EOs in their complexity for environmentally friendly treatments to control mycotoxigenic and plant pathogen fungi. To provide lasting effects, it would be interesting to develop formulae protecting the EOs from alterations and favoring a progressive release within the environment of the targeted fungi. Confirming the activity of *A. pusilla* EO on a diversity of *F. avenaceum* strains and other *Fusarium* species producing ENNs would be necessary before proposing the practical use of this natural production for controlling ENNs in cereal-derived products.

## Figures and Tables

**Table 1:** Published article with data on the effect of essential oils (EOs) on the mycelial growth of *F. avenaceum*.

Plants	Origin	Extraction Yield	Major Components **	Methods ***	Inhibition of Growth	Reference
*Abies sibirica*	Com. */Lithuania	-	-	APD, 10 µL pure EO, (27 °C, 5 d)	11 mm diameter	[17]
*Asarum heterotropoides*	Roots and rhizomes/China	1.6%	Methyleugenol (38.7–43.8%), Safrol (12.9–15.3%)	BD, (26 °C, 72 h)	MIC50 = 0.61 mg·mL^−1^	[18]
*Carum carvi*	Com./Lithuania	-	-	APD, 10 µL pure EO, (26 °C, 72 h)	89 mm diameter	[17]
Cinnanom	Com./Pakistan	-	Eugenol (75.5%)	AD, (20 °C, 10 d)	MIC = 0.5 µL·mL^−1^	[16]
Cinnanom	Com./Pakistan	-	Eugenol (75.5%)	Volatiles, 10 µL EO, Agar medium, (20 °C, 6 d)	84% Inhibition	[16]
Citronella	Com./Pakistan	-	Geraniol (53.6%)	AD, (20 °C, 10 d)	MIC = 0.5 µL·mL^−1^	[16]
Citronella	Com./Pakistan	-	Geraniol (53.6%)	Volatiles, 10 µL EO, Agar medium, (20 °C, 6 d)	84% Inhibition	[16]
*Citrus paradis* Grapefruit	Com./Germany	-	Linalyl acetate (1.87%), α-Terpineol (1.83%), Nootkatone (1.37%)	AD, 2% EO, (25 °C, until control covered plates)	12% Inhibition	[19]
Clove	Com./Pakistan	-	Eugenol (80.9%)	AD, (20 °C, 10 d)	MIC = 0.5 µL·mL^−1^	[16]
Clove	Com./Pakistan	-	Eugenol (80.9%)	Volatiles, 10 µL EO, Agar medium, (20 °C, 6 d)	86% Inhibition	[16]
*Cymbopogon citratus* (Lemongrass)	Com./Germany	-	α and β-Citral (68.9%)	AD, 2% EO, (25 °C, until control covered plates)	MIC = 0.12 µL·mL^−1^	[19]
*Eucalyptus* *globus*	Com./Lithuania	-	-	APD, 10 µL pure EO, (27 °C, 5 d)	28 mm diameter	[17]
*Hyssopus* *officinalis*	Fresh flowering tops/Italy	2.3 mL·kg^−1^	Pinocamphone (34%), β-Pinene (10.5%), α-phellandrene (7.4%)	AD, (22 °C, 7 d)	MIC = 1.5 mg·mL^−1^	[20]
*Hyssopus* *officinalis*	Fresh flowering tops/Italy	3.1 mL·kg^−1^	Isopinocamphone (29%), Pinocamphone (18.5%), β-Pinene (10.8%)	AD, (22 °C, 7 d)	MIC = 1.2 mg·mL^−1^	[20]
*Lavandula* *hybrida*	Com./Lithuania	-	-	APD, 10 µL pure EO, (27 °C, 5 d)	12 mm diameter	[17]
Lemon	*Citrus* rind/Nigeria	-	α-Terpineol (31.2%), l-Limonene (14.4%), β-Pinene (12.4%)	AWD, (25 °C, 48 h)	MIC = 100 mg·mL^−1^	[21]
Lime	*Citrus* rind/Nigeria	-	d-Limonene (27.8%), β-Pinene (25.2%),	AWD, (25 °C, 48 h)	MIC = 100 mg·mL^−1^	[21]
Lemongrass	Com./Pakistan	-	Geranial (40.8%)	AD, (20 °C, 10 d)	MIC = 0.5 µL·mL^−1^	[16]
Lemongrass	Com./Pakistan	-	Geranial (40.8%)	Volatiles, 10 µL EO, Agar medium, (20 °C, 6 d)	84% Inhibition	[16]
*Luppia* *javanica*	Com./USA	-	α and β-Citral (36.0%), α-Terpineol (18.3%)	AD, 2% EO, (25 °C, until control covered plates)	MIC = 0.12 µL·mL^−1^	[19]
*Litsea cubeba*	Com./Germany	-	α and β-Citral (68.9%)	AD, 2% EO, (25 °C, until control covered plates)	MIC = 0.12 µL·mL^−1^	[19]
*Melaleuca* *alternifolia*	Com./Germany	-	α-Terpineol (6.88%), Cadinene (3.86%), β-Gurjunene (1.23%)	AD, 2% EO, (25 °C, until control covered plates)	MIC = 0.5 µL·mL^−1^	[19]
*Melaleuca* *alternifolia*	Com./Lithuania	-	-	APD, 10 µL pure EO, (27 °C, 5 d	22 mm diameter	[17]
*Melaleuca* *leucadendron*	Com./Germany	-	α-Terpineol (36.57%), Caryophyllene (2.60%), α-Caryophyllene (1.70%)	AD, 2% EO, (25 °C, until control covered plates,)	MIC = 0.1 µL·mL^−1^	[19]
*Mentha* *pulegium*	Fresh aerial parts/Tunisia	1.84%	Menthol (39.2%), 1,8-Cineole (17.1%),	AD, 0.5 mg EO mL^−1^, (24 °C, 7 d)	32.5% Inhibition	[22]
*Mentha* *spicata*	Com./USA	-	-	AWD, 100 µL pure EO, (18 °C, 7 d)	100% Inhibition	[23]
*Monathotaxis* *littoralis*	Fresh leaves/Uganda	1.97%	-	APD, 10 µL EO, (25 °C, 7–10 d):	MIC = 103 mg·mL^−1^	[24]
Oregano	Dried leaves/Algeria	-	Carvacrol (59.03%)	AD, (25 °C, 72 h)	MIC = 0.078 µL·mL^−1^	[25]
Oregano	Com./Pakistan	-	Carvacrol (63.8%)	AD, (20 °C, 10 d)	MIC = 0.125–0.25 µL·mL^−1^	[16]
Oregano	Com./Pakistan	-	Carvacrol (63.8%)	Volatiles, 10 µL EO, Agar medium, (20 °C, 6 d)	100% Inhibition	[16]
*Origanum vulgare*	Air-dried leaves, 21 accessions/Lithuania	-	Sabinene (0.3–25.1%), β-Caryophyllene (4.7–20.1%), Germacrene D (2.1–20.1%), Caryophyllene oxide (0.7–24.4%)	AWD, 100 µL 0.5% EO, (18 °C, 7 d)	3.8–19.5 mm diameter	[25]
*Origanum vulgare*	Air-dried inflorescences, 21 accessions/Lithuania	-	Sabinene (0.9–18.3%), β-Caryophyllene (5.4–24.5%), Germacrene D (1.5–12.2%), Caryophyllene oxide (0.7–24.4%)	AWD, 100 µL 0.5% EO, (18 °C, 7 d)	9.9–22 mm diameter	[25]
*Origanum vulgare*	Fresh aerial parts/Tunisia	0.90%	Thymol (29.6%), *p*-Cymene (29.4%)	AD, 0.5 mg EO mL^−1^, (24 °C, 7 d)	77.4% Inhibition	[22]
Palmarosa	Com./Pakistan	-	Geraniol (72.26%)	AD, (20 °C, 10 d)	MIC = 1.0 µL·mL^−1^	[16]
Palmarosa	Com./Pakistan	-	Geraniol (72.26%)	Volatiles, 10 µL EO, Agar medium (20 °C, 6 d)	87% Inhibition	[16]
*Pimpinella* *anisum*	Com./Lithuania	-	-	APD, 10 µL pure EO, (27 °C, 5 d)	30 mm diameter	[17]
*Pinus* *halepensis*	Fresh needles/Tunisia	0.30–0.87%	(*Z*)-Caryophyllene (16–28.9%), β-Myrcene (8.5–22.9%), α-Pinene (11.7–13.14%)	AD, 4 µL EO mL^−1^, (25 °C, 7 d)	41.9–51.8% Inhibition	[26]
*Pinus pinea*	Fresh needles/Tunisia	0.40%	Limonene (54.1%),	AD, 4 µL EO mL^−1^, (25 °C, 7 d)	61.1% Inhibition	[27]
*Piper capense*	Fresh whole plant/Kenya	0.20%	δ-Cadinene (16.82), β-Pinene (7.24%), β-Bisabolene (5.65%),	APD, 10 µL EO, (25 °C, 7–10 d)	MIC = 66.3 mg·mL^−1^	[28]
*Pistacia lentiscus*	Fresh leaves/Tunisia	0.14%	α-Pinene (20.6%), Limonene (15.3%), β-Pinene (9.6%)	AD, 4 µL EO mL^−1^ (25 °C, 7 d)	44.4% Inhibition	[29]
*Pistacia terebintus*	Fresh leaves/Tunisia	0.24%	α-Terpinene (41.3%), α-Pinene (19.2%)	AD, 4 µL EO mL^−1^, (25 °C, 7 d)	68.0% Inhibition	[29]
*Pistacia vera*	Fresh leaves/Tunisia	0.27%	α-Terpinene (32.4%), Limonene (25.1%)	AD, 4 µL EO mL^−1^, (25 °C, 7 d)	63.2% Inhibition	[29]
*Rosmarinus* *officinalis*	Fresh aerial parts/Tunisia	0.60%	1,8-Cineole (40.9%),α-Pinene (24.2%),	AD, 0.5 mg EO mL^−1^, (25 °C, 7 d)	21.8% Inhibition	[22]
*Syzygium* *aromaticum*	Com./Lithuania	-	-	APD, 10 µL pure EO (27 °C, 5 d)	90 mm diameter	[17]
Thyme	Dried leaves/Algeria	0.85%	Thymol (46.97%), Linalool (3.94%)	AD, (25 °C, 72 h)	MIC = 0.156 µL·mL^−1^	[25]
Thyme	Com./Pakistan	-	Linalool (64.0%)	AD, (20 °C, 10 d)	MIC = 0.5 µL·mL^−1^	[16]
Thyme	Com./Pakistan	-	Linalool (64.0%)	Volatiles, 10 µL EO, Agar medium (20 °C, 6 d)	100% Inhibition	[16]
*Thymus* *capitatus*	Fresh aerial parts/Tunisia	2.85%	Carvacrol (69.15%)	AD, 0.5 mg EO mL^−1^, (24 °C, 7 d)	89.9% Inhibition	[22]
*Thymus* *capitatus*	Fresh aerial parts/Tunisia	1.9 to 3.15%	Carvacrol (69.69 to 83.86%)	AD, 0.4 µL EO mL^−1^, (25 °C, 7 d)	4 to 93% Inhibition	[30]
*Thymus* *pilegioides*	Com./Lithuania	-		APD, 10 µL pure EO, (27 °C, 5 d)	33 mm diameter	[17]
*Thymus* *vulgaris*	Com. Austria	-	Thymol (45.75%), Limonene (15.15%),	AD, 2% EO, (25 °C, until control covered plates)	MIC = 0.025 µL·mL^−1^	[19]
*Vepris* *macrophylla*	Leaves/Madagascar	-	Citral (56.3%)	AD, (22 °C, 6 d)	MIC = 130.4 µg·mL^−1^	[31]

* Com. = commercial EO. ** Three first abundant components, or sum of components reaching 50%. *** Abbreviations of methods: AD = agar diffusion, APD = agar and impregnated paper disc, AWD = agar well diffusion, BD: broth dilution.

**Table 2:** Extraction yield of essential oils from eight Tunisian aromatic plants.

Plant	EO Extraction Yield (%)
*Ammoides pusilla* (AP2)	1.64
*Thymus capitatus* (TC)	2.45
*Carum carvi* (CC)	1.57
*Origanum vulgare* (OV)	0.46
*Myrtus communis* (MC)	0.45
*Artemisia absintum* (AA)	0.96
*Schinus terbentofonius* (ST)	2.50
*Mentha spicata* (MS)	1.10

**Table 3:** Chemical composition (%) of essential oils of *A. pusilla* using GC-MS.

Compounds	RT ^1^	% in AP1	% in AP2
* **Monoterpene hydrocarbons** *			
α-thujene	5.69	0.37%	0.24%
α-pinene	5.86	0.21%	0.36%
Sabinene	6.76	0.57%	----
β-myrcene	7.12	0.48%	0.30%
α-terpinene	7.80	----	0.19%
*p*-Cymene	8.00	19.89%	14.59%
α-thujene	5.69	0.37%	0.24%
γ-terpinene	8.89	27.03%	16.82%
*o*-Allyltoluene	9.72	0.25%	----
* **Oxygenated monoterpenes** *			
Borneol	11.86	0.18%	----
Terpinen-4-ol	12.17	0.67%	0.58%
Thymol methyl ether	13.56	9.18%	8.07%
Benzene, 2-methoxy-4-methyl-1-(1-methylethyl)	13.69	0.73%	0.52%
Thymol (isomere)	15.08	1.70%	2.88%
Thymol	15.26	34.70%	53.55%
NI	15.36	0.25%	0.29%
Carvacrol	15.59	0.41%	0.68%
2-(*t*-butyl)-5,6-dihydro-4*H*-cyclopenta[b]thiophene	17.74	0.61%	0.53%
1-Methoxy-2-tert-butyl-6-methylbenzene	18.87	2.48%	0.20%
Identified compounds		97.27%	99.51%
Monoterpene hydrocarbons		48.83%	32.70%
Oxygenated monoterpenes		47.82%	66.58%
Extraction yield		1.6%	1.64%

^1^ RT: Retention time; AP1 = EO of *A. pusilla* obtained from the batch of plant 1. AP2 = EO of *A. pusilla* obtained from the batch of plant 2. Calculated percentage (%) of each identified compound is based on the total detected compounds.

**Table 4:** Linear growth index of *F. avenaceum* and ENNs accumulation in presence of different concentrations of AP1 EO.

	Concentrations of Essential Oil (mL·L^−1^)					
	0 (Control)	0.1	0.25	0.5	0.75	1
Linear growth index	16.22	10.65	3.34	0.19	0.00	0.00
µg enniatins/cm^2^ mycelium	2.06	0.72	0.49	0.00	0.00	0.00

Mycelial areas were measured every day for 7 days.

**Table 5:** Linear growth index of *F. avenaceum* and ENNs accumulation after 10 days in presence of different quantities of *A. pusilla* EO (AP1) volatile components.

	µL EO L^−1^ Air				
	0 (control)	3.3	6.7	10	16.7
Linear growth index	16.02	15.68	12.11	10.10	2.97
% growth inhibition at 10 d	0	21.97	72.83	98.40	100
µg ENNs cm^−2^ mycelium (10 d)	1.34	0.44	0.26	<LOQ *	<LOQ

* LOQ = limit of quantification.

## Data Availability

The raw data associated to the data presented in this study are available on request from the corresponding author.

## Sample Availability

Fungal strains, samples of plant material and tested EOs are available from authors.
